# Pan-cancer evaluation of gene expression and somatic alteration data for cancer prognosis prediction

**DOI:** 10.1186/s12885-021-08796-3

**Published:** 2021-09-25

**Authors:** Xingyu Zheng, Christopher I. Amos, H. Robert Frost

**Affiliations:** 1 Department of Biomedical Data Science, Geisel School of Medicine, Dartmouth College, Hanover, NH USA; 2 Department of Medicine, Institute for Clinical and Translational Research, Baylor College of Medicine, Houston, TX USA

**Keywords:** Cancer prognosis prediction, Multi-omics data, Pathway analysis, L1 penalized regression model

## Abstract

**Background:**

Over the past decades, approaches for diagnosing and treating cancer have seen significant improvement. However, the variability of patient and tumor characteristics has limited progress on methods for prognosis prediction. The development of high-throughput omics technologies now provides multiple approaches for characterizing tumors. Although a large number of published studies have focused on integration of multi-omics data and use of pathway-level models for cancer prognosis prediction, there still exists a gap of knowledge regarding the prognostic landscape across multi-omics data for multiple cancer types using both gene-level and pathway-level predictors.

**Methods:**

In this study, we systematically evaluated three often available types of omics data (gene expression, copy number variation and somatic point mutation) covering both DNA-level and RNA-level features. We evaluated the landscape of predictive performance of these three omics modalities for 33 cancer types in the TCGA using a Lasso or Group Lasso-penalized Cox model and either gene or pathway level predictors.

**Results:**

We constructed the prognostic landscape using three types of omics data for 33 cancer types on both the gene and pathway levels. Based on this landscape, we found that predictive performance is cancer type dependent and we also highlighted the cancer types and omics modalities that support the most accurate prognostic models. In general, models estimated on gene expression data provide the best predictive performance on either gene or pathway level and adding copy number variation or somatic point mutation data to gene expression data does not improve predictive performance, with some exceptional cohorts including low grade glioma and thyroid cancer. In general, pathway-level models have better interpretative performance, higher stability and smaller model size across multiple cancer types and omics data types relative to gene-level models.

**Conclusions:**

Based on this landscape and comprehensively comparison, models estimated on gene expression data provide the best predictive performance on either gene or pathway level. Pathway-level models have better interpretative performance, higher stability and smaller model size relative to gene-level models.

**Supplementary Information:**

The online version contains supplementary material available at 10.1186/s12885-021-08796-3.

## Background

Over the past decades, considerable progress has been achieved in diagnosing and treating cancer, with the overall cancer death rates between 1999 and 2015 decreasing by 1.8% per year for men and 1.4% per year for women [1]. However, the variability of patient and tumor characteristics has limited progress on methods for prognosis prediction, despite significant efforts by members of the cancer research community [2, 3]. Several prognostic models for cancer patients using clinical and pathological variables have been developed and widely used in clinical oncology practice [4–6]. With the development of microarrays to detect molecular profiles of patients, some multi-gene assays have been designed and successfully applied in clinical care, such as the assays for prediction of breast cancer recurrence [7, 8]. The development of high-throughput technologies now enables the integration of large-scale molecular profiling data for developing cancer prognostic tools, e.g., RNA profiling through arrays or sequencing enables the measurement of gene-level expression [9], DNA sequencing enables the calling of somatic mutations [10] and application of SNP arrays enable the detection of copy number variation [11]. Many gene-level prognostic models based on gene expression data have been published [12–15], copy number variation has provided insights for cancer prognosis prediction [16, 17], and somatic mutations are often reliably associated with cancer prognosis [18–21]. Given the high level of stochastic variation found in the measures of individual genes, various studies have focused on developing pathway-level models for cancer prognosis prediction [22–25]. A limitation of some single-omics prognostic models is that a single type of genomic measurement may be insufficient to characterize fully the features that lead to cancer progression.

Over the past decade, several large repositories, such as The Cancer Genome Atlas (TCGA) [26] and The International Cancer Genome Consortium (ICGC) [27], have been developed to collect comprehensive multi-omics data on a large group of cancer patients spanning the most common types of human cancer. In TCGA, tumor and normal samples from over 6000 patients have been profiled, covering 37 types of genomic and clinical data for 33 cancer types. Studies based on the analysis of TCGA data range from the comprehensive analysis of specific cancers to more comprehensive landscapes across the most common cancer types. The development of these repositories offers extraordinary opportunities to integrate multi-omics data and researchers have noted that accurate modeling of cancer biology requires multidimensional genomic measurements [28–31]. Several recent studies have focused on integration of multi-omics data, especially for survival analysis. For example, studies such as [32–35] have integrated copy number variation and gene expression, and [36–39] have integrated somatic mutation and gene expression. Although a large number of studies have explored the integration of multi-omics data for cancer prognosis prediction [25, 28, 29, 34, 36–40], there still exists a gap of knowledge regarding the prognostic landscape across multi-omics data for multiple cancer types and both gene-level and pathway-level models. In this study, we systematically evaluate three types of omics data (gene expression, copy number variation and somatic point mutation) covering both DNA-level and RNA-level features. We construct the landscape of predictive performance using these three types of omics data for 33 cancer types on both the gene and pathway levels. Based on this landscape, we highlight the cancer types and omics modalities that support the most accurate prognostic models.

## Methods

### Data sources

TCGA data were accessed via the UCSC Xena data hub [41]. In all, 33 cohorts listed in Supplementary Table X1 were retained for analysis, which included 30 different cancer types and 3 combinations of cancer subtypes; 4 cancer cohorts were excluded because of an insufficient number of samples (Bile Duct Cancer cohort, Formalin Fixed Paraffin-Embedded Pilot Phase II cohort, Large B-cell Lymphoma cohort and Uterine Carcinosarcoma cohort). We downloaded and analyzed gene expression (GE) RNA-seq data, gene-level copy number variation (CNV) data, gene-level non-silent somatic point mutation (SPM) data and survival data for these 33 cancer type cohorts. We focused on the overall survival (OS) end point as the prognostic outcome. Overall survival (OS) is the gold standard primary end point since OS is universally recognized as being unambiguous, unbiased and clinical relevant [42].

For the pathway definitions, we adopted the Hallmark pathway collection from the Molecular Signatures Database (MSigDB) version 6.2 [43]. The Hallmark pathways were generated by a hybrid approach combining computation with manual expert curation and can reduce redundancy and produce more robust enrichment analysis results. The Hallmark pathway collection of MSigDB consists of 50 gene sets derived by aggregating and clustering all other MSigDB gene sets, followed by assignment of well-defined biological states or processes and refinement of genes relevant to the corresponding biological theme [44].

### Prognostic models

**Fig. 1:**
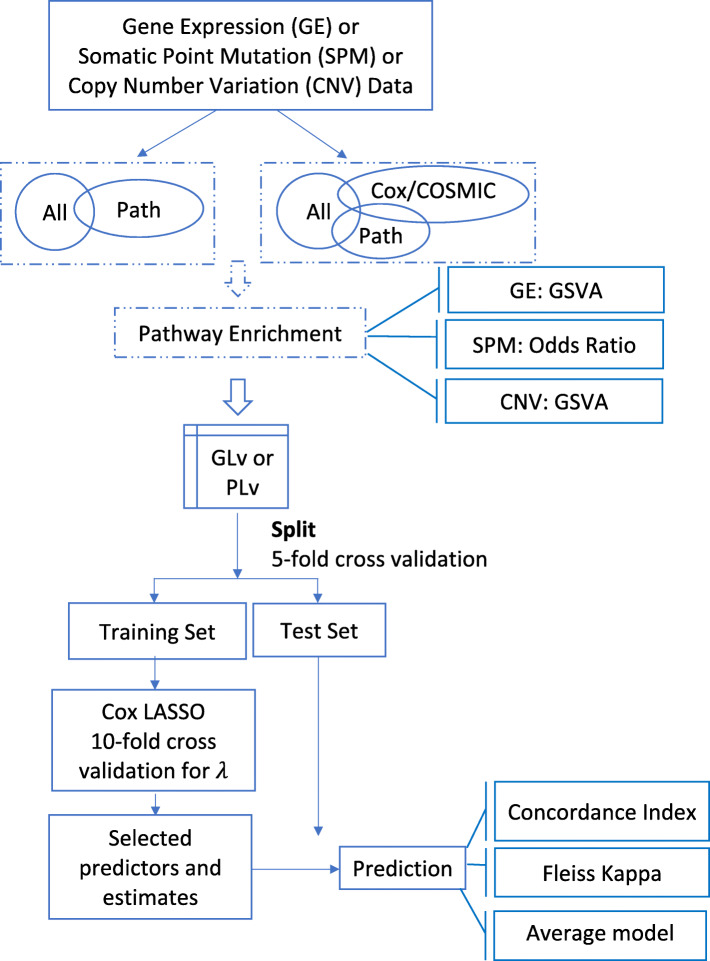
Workflow of gene-level and pathway-level models. Gene-level data matrix of GE/SPM/CNV is input into the workflow. Genes are pre-filtered either by intersecting with the pathway collection (shown as ‘Path’) or further filtering the genes by intersecting with COSMIC genes (shown as ‘COSMIC’) or significant genes (*p*-value less than 0.05) in univariable Cox models (shown as ‘Cox’). Then, for the pathway-level models, gene set enrichment is conducted to transform the gene-level matrix into a pathway-level matrix. For GE and CNV data, GSVA is applied and for SPM, odds ratio is applied to conduct gene set enrichment. While for the gene-level models, this step is skipped. With the filtered gene-level data matrix or the transformed pathway-level data matrix as the predictor matrix, we conducted nested cross validation to test the predictive performance of gene-level and pathway-level models. A 5-fold cross validation separates the data into training and test sets. In the training set, a Lasso (least absolute shrinkage and selection operator) or L1-penalized Cox model is fit with the shrinkage parameter chosen by a nested 10-fold cross validation. With the selected predictors and coefficient estimates, the estimated model is applied to the test set and three metrics are adopted to measure the prediction: i) the predictive performance is measured by the concordance index, ii) the model robustness is measured by Fleiss Kappa, iii) the model parsimony is measured by average model size

In this study, we used penalized Cox proportional hazards models with either gene-level or pathway-level predictors as the prognostic models. Our workflow for both the gene-level and pathway-level models is illustrated in Fig. 1.

As shown in Fig. 1, we first conducted filtering on the gene list. To make a fair comparison between gene-level and pathway-level models, we restricted the genes to include only the genes that are present in the Hallmark pathway collection. In addition to this filtering, we also evaluated the prognostic accuracy of models after further filtering the genes by either intersecting with COSMIC (The Catalogue Of Somatic Mutations In Cancer) genes [45] or significant genes (*p*-value less than 0.05) in univariable Cox models. To avoid an overfitting bias when filtering according to univariable Cox models, the models were estimated on the training set.

After the filtering step, for the pathway-level model, we conducted single sample gene set enrichment to calculate the sample-level pathway scores and transform the sample-by-gene data matrix into a sample-by-pathway data matrix. For the GE data, we adopted the GSVA (Gene set variation analysis) method [46]. GSVA is an unsupervised and sample-wise gene set enrichment method designed for gene expression data, which calculates a score indicating pathway activity for each sample and pathway. GSVA generates probability density estimates for each gene, which protects it against systematic gene-specific biases and brings distinct profiles to a common scale. Considering the rationale of GSVA and the similar structure of GE and TCGA level 3 CNV data (both are gene-level continuous data), we directly applied GSVA to the CNV data. Since the SPM data is binary, we computed sample-level pathway scores using a log-odds ratio method. Specifically, for each pathway and sample, we created a two-by-two table counting the number of genes according to the presence of somatic point mutations and pathway membership. To avoid the 0 count in the two-by-two table, we added 0.5 to each of the cells (known as Haldane-Anscombe correction [47, 48]). Haldane-Anscombe correction is a common practice, which also removes some bias from the estimator. Using this table, an odds ratio is calculated to indicate the association between pathway membership and mutation status and the log of this odds ratio is used as the sample-level pathway score.

Then, with the filtered gene-level data matrix or the transformed pathway-level data matrix as the predictor matrix, we conducted cross validation to test and compare the predictive performance of gene-level and pathway-level models. Specifically, we conducted 5-fold cross validation of a Lasso-penalized [49] Cox model with the shrinkage parameter chosen by a nested 10-fold cross validation. The Lasso-penalized Cox model was implemented using the functions ‘cv.glmnet()’ and ‘glmnet()’ in the R package ‘glmnet’ [50] with default parameter values.

### Integrative models

In this study, we also evaluated the integration of multi-omics data for cancer prognosis prediction. For the integrated analysis, we evaluated two integration methods. In the first method, we combined the data matrices for each omics modality into a single predictor matrix and then performed cross validation as detailed above. The predictor standardization was implemented by default in glmnet to bring different types of variables to the same scale. In the second method, we explored the use of Group Lasso [51] to integrate multi-omics data. Group Lasso is an extension of Lasso for data with a group structure. The principle of Group Lasso is that the variables in the same group should be either all included or all discarded. In this study, for each gene or pathway, we have scores for GE, CNV and SPM separately. Each gene or pathway can function as a group in the Group Lasso with its GE, CNV and SPM variables as group members, which indicate three dimensions of each gene or pathway and may capture a similar biological association with cancer prognosis. After model estimation using a Group Lasso penalty using the function ‘cv.grpsurv()’ in the R package ‘grpreg’ [52], all the GE, CNV and SPM variables in the remaining non-zero groups were included into the prognostic models.

### Model evaluation metrics

The concordance index (CI), or c-index, is one of the most widely used metrics for survival models and can be interpreted as the measurement of concordance between the predicted and true survival outcomes with a value of 1 indicating perfect prediction and a value of 0.5 indicating random prediction [53]. In our study, we used the average concordance index across cross validation replications to quantify the predictive performance of each model. Inter-rater reliability represents the ability of a model to assign the same score to the same variable for different repeated raters [54]. The Fleiss kappa statistic is widely used to test inter-rater reliability and can be interpreted as the measurement of agreement among different replications with a value of 1 indicating perfect agreement and a value equal to or less than 0 indicating no agreement. In our study, we used the Fleiss kappa statistic [55] to evaluate the repeatability and inter-rater reliability among replications. Specifically, each trained model is a rater that is assigning each variable (gene or pathway) to either being included or excluded in the model. Finally, we used the average number of predictors retained in the trained models to measure model parsimony.

## Results

**Fig. 2:**
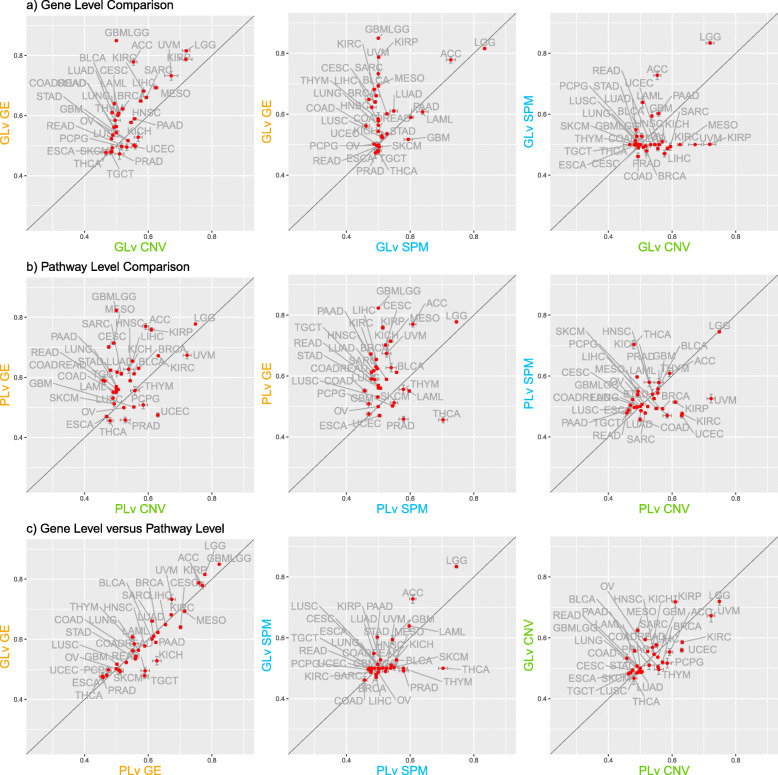
The comparative results for both gene-level and pathway-level prognostic models estimated using GE, SPM and CNV data from multiple cancer types. ‘PLv’ represents ‘pathway-level’ and ‘GLv’ represents ‘gene-level’. The dots represent the values of the concordance index and the bars represent the standard error

Figure 2 displays the results for both gene-level and pathway-level prognostic models estimated on GE, SPM and CNV data from 33 TCGA cancer types. Rows a and b show that models estimated on GE data most often provide the best predictive performance using either gene-level or pathway-level predictors. The comparison of pathway-level and gene-level GE models in row c shows that these models have similar predictive power. For CNV and SPM data, the gene-level models perform slightly better than the pathway-level models.

Row c in Fig. 2 shows the predictive power of single-omics data on both the gene and pathway level. Focusing on cohorts with concordance index values larger than 0.7, which indicates good model performance and is widely used as a standard in the literature [56, 57], GE-based models can predict well for LGG, GBMLGG, KIRP, ACC, MESO on both levels, CESC on the pathway level and UVM on the gene level; SPM-based models can predict well for LGG and ACC on the gene level and THCA on the pathway level; CNV-based models can predict well for LGG and UVM on the pathway level and KIRP on the gene level.

**Fig. 3:**
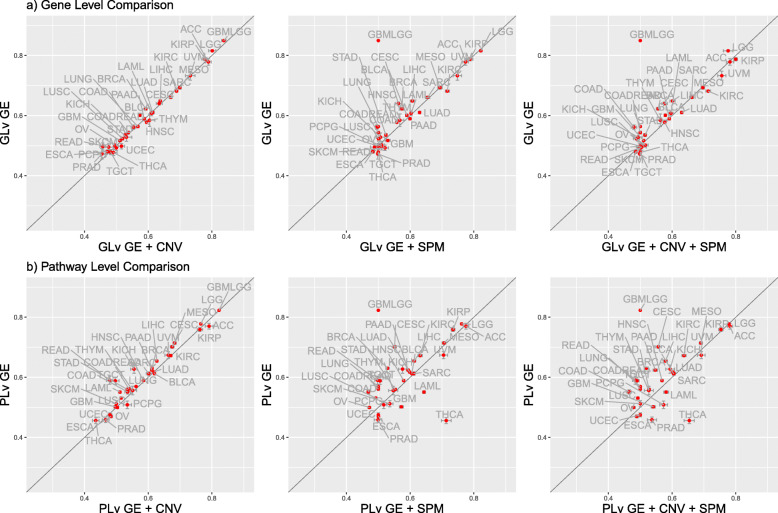
Comparative results of adding SPM or CNV data to GE data. ‘PLv’ represents ‘pathway-level’ and ‘GLv’ represents ‘gene-level’. The dots represent the values of the concordance index and the bars represent the standard error

Based on the results shown in Fig. 2, survival models estimated using GE data most often have the best predictive performance among all single omics models. Given this, we next investigated whether the integration of SPM or CNV data with GE data could improve performance over models based on just GE data. Figure 3 displays the comparative results of GE data alone versus integration of GE with SPM or CNV data on both the gene and pathway levels. These results show that, in general, adding CNV or SPM data to GE data does not improve predictive performance. This is consistent with findings from Zhao et al. [28]. Adding CNV data to GE data neither increases nor decreases the prediction relative to GE data alone, on both the gene and pathway levels. A similar result is obtained by adding SPM data to GE data on the gene level. The additional SPM data to GE data on the pathway level increases predictive power for cohorts such as THCA but decreases performance for cohorts such as CESC. It is worth noting that, for THCA cohort, the integration of pathway-level SPM and GE data does not perform better than the SPM-only model. Therefore, for THCA cohort, the SPM-only models are optimal. We then investigated the prognostic predictors used in the THCA pathway-level SPM model and found that the MSigDB Hallmark Glycolysis and Spermatogenesis pathways were included as predictors in more than 95% of the estimated models. The biological association of these two pathways to thyroid cancer has been detailed by other researchers. The thyroid gland, previously assumed to not have an impact on spermatogenesis and male fertility, is now recognized to have an important role in male reproductive functions [58, 59]. A considerable amount of data shows that thyroid hormone influences steroidogenesis as well as spermatogenesis [60]. And it is reported that glycolysis-related proteins, such as LDHA, are associated with invasiveness and prognosis of thyroid cancer [61].

**Fig. 4:**
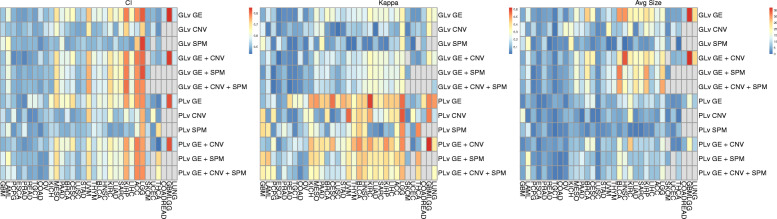
Heatmap of concordance index, Fleiss Kappa statistics and average model size across cohorts and models. ‘PLv’ represents ‘pathway-level’ and ‘GLv’ represents ‘gene-level’. The cells in grey represent models that cannot converge and in this case, no predictors could be selected to predict prognosis

In addition to predictive performance, other features such as model robustness and parsimony are also important metrics for the evaluation cancer prognosis prediction models. We utilized the concordance index (CI) to evaluate prediction, Fleiss Kappa statistics to evaluate model robustness and average model size to evaluate parsimony. Figure 4 includes heatmaps that illustrate the pattern of these three metrics across all cohorts and models.

The CI heatmap in Fig. 4 shows that the predictive performance is cancer dependent. This result is concordant with findings reported in Jardillier et al. [62]. For the cohorts located in the left area of the CI heatmap, all evaluated models have poor prognostic power. These models are also associated with lower Kappa values and smaller model sizes as shown in the other two heatmaps, indicating that these models tend to select a small set of random predictors thus the prediction is poor and the models are unstable. For the cohorts located in the right portion of the CI heatmap, the evaluated models had relatively good predictive performance. For these cohorts, the Kappa heatmap indicates that the pathway-level models have higher Kappa values, which indicates better robustness across multiple cross validation splits and replications. As shown in the average model size heatmap, the pathway-level models for these cohorts are also more parsimonious. Overall, these results demonstrate that the pathway-level models have the advantages of better interpretation, higher stability and smaller model size across multiple cancer types and omics data types.

In addition to the models above, we also investigated two different approaches for filtering genes before model estimation. The first filtering approach we evaluated retained only those genes with a significant *p*-value in a univariable Cox model fit on the training set during cross validation. Supplementary Figure S1 displays the predictive performance achieved by this filtering approach relative to models estimated without gene filtering. As shown in this figure, filtering genes with a univariable Cox model failed to improve predictive performance for gene-level models but did improve performance for pathway-level GE and CNV models. For pathway-level model estimated using SPM data, however, filtering resulted in a model without any pathway-level predictors at the optimal Lasso penalization threshold (the relative performance for this model is therefore not included in Supplementary Figure S1). The failure of the filtered SPM pathway-level model to retain predictors after Lasso penalization may be due to the fact that the SPM data itself is sparse and binary and that, after filtering, too few genes are retained to accurately estimate single sample pathway scores. In this case, it is likely that the pathway-level variables contain insufficient information to predict cancer prognosis. Surprisingly, filtering genes based on the results from univariable Cox models did not improve predictive performance for either gene-level or pathway-level multi-omic models. Supplementary Figure S2 row a displays the comparative results of gene filtering for these integration models. The second type of filtering we investigated was limited to the SPM-based models and it filtered the genes according to the COSMIC database. Specifically, we removed any genes without a known cancer association according to COSMIC. As shown in Supplementary Figure S2 row b, COSMIC-based filtering failed to improve predictive performance for either the gene-level or pathway-level models.

In addition to gene filtering, we also investigated the use of a Group Lasso penalty for multi-omics models and the incorporation of clinical stage as a predictor. As illustrated in Supplementary Figure S2 row c the use of a Group Lasso penalty did not improve the predictive performance for the multi-omics models. Supplementary Figure S3 illustrates the impact of adding clinical stage to the models. Surprisingly, adjusting for clinical stage failed to improve the predictive performance for expression data and only weakly improved predictive accuracy for selected CNV or SPM prognostic models. This finding suggests that gene expression levels and clinical staging are correlated, so that little is gained by adding stage information to models for expression data. Other factors that may be driving this result include: i) insufficient samples for many TCGA cohorts to achieve good results via stage-based stratification and five-fold cross validation, and ii) the fact that some cancer types in TCGA represent stage-specific subtypes, such as the LGG and GBM cohorts.

## Discussion

In this study, we construct the prognostic landscape using three types of omics data for 33 cancer types on both the gene and pathway levels. Based on this landscape, we found that predictive performance is cancer type dependent and that, relative to gene-level models, pathway-level models have better interpretative performance, higher stability and smaller model size across multiple cancer types and omics data types. We also highlight the cancer types and omics modalities that support the most accurate prognostic models. Beyond this landscape, we evaluated the impact of other modeling parameters including gene filtering, integrative methods and adjustment of clinical stage. In general, models estimated on GE data provide the best predictive performance on either gene or pathway level and adding CNV or SPM data to GE data does not improve predictive performance. Although adding CNV or SPM data into the GE models did not on average improve the predictive power significantly on the pathway level, as shown in the Supplementary Figure S4, the pathway level variables of CNV and SPM still contributed to risk prediction for some models. In the pathway-level integrative model of GE and CNV, the average proportion of CNV variables across all cohorts is 0.49 and for ESCA and UCEC, the proportions are even larger than 0.70. In the pathway-level integrative model of GE and SPM, the average proportion of SPM variables across all cohorts is 0.42 and for ESCA, GBM, PRAD and LUSC, the proportions are even larger than 0.70. Compared with the average proportions of 0.18 and 0.25 respectively in the gene-level integrative models, this finding implies that pathway-level models may exploit more information from CNV and SPM data than gene-level models.

Among the cohorts with concordance index values above 0.7, LGG, ACC and THCA are noteworthy. The LGG cohort performs better than all other cohorts with strong predictive power, robustness across replications and relatively parsimonious models. For the LGG cohort, all 6 models have high concordance index values above 0.7. As shown in the CI heatmap in Fig. 4, the LGG cohort performed remarkably well for all models with the gene-level CNV model having the worst predictive performance (CI is 0.72) and gene-level SPM having the best predictive performance (CI is 0.83). This implies that effective prognostic performance for this cohort can be achieved without gene expression data. Specifically, the LGG SPM models have equivalent performance as the LGG GE models on both the pathway-level and gene-level. Equivalent predictive performance results have also been reported in Zheng et al. [63]. While the gene-level LGG CNV model is slightly worse than the GE and SPM models, the pathway-level LGG CNV model works as well as the GE and SPM models. For the ACC cohort, only the GE and SPM-based models work well using gene-level predictors. For models estimated using pathway-level predictors, only those based on GE data work well for the ACC cohort. Gene expression data is therefore not required to generate effective predictive models for ACC. For the THCA cohort, it is surprising that among all 6 models, only the pathway-level SPM model can predict well. This result may be due to the fact that thyroid cancer has a very low death rate (0.03 in the TCGA data), which makes estimation of survival models challenging.

The underlying factors leading to the heterogenous predictive performance for different cohorts are unknown. These cohorts, located in the left portion in the CI heatmap in Fig. 4, have variable death rates, ranging from 0.02 to 0.75, and variable sample sizes, ranging from 80 to 500. For these cohorts, these three omics data types could not predict prognosis and other characteristics beyond the scope of this study may dominate prognosis, such as clinical variables specific to each cancer type, more accurate characteristics of each sample and even more accurate measurement of each tumor cell with the high-speed development of single-cell sequencing technology.

Although our study was conducted with the Hallmark pathway collection and OS end point as justified in the Data sources section, our method can be extended to other pathway collections in the MSigDB database and other end points including Disease-Specific Survival (DSS), Disease-Free Interval (DFI) and Progression-Free Interval (PFI) in UCSC Xena datahub. We have conducted the basic analysis of an alternative survival outcome, disease free interval (DFI). We explored GE, CNV and SPM data for DFI prediction on both the gene and pathway level. Supplementary Figure S5 shows the predictive power of single-omics data on both the gene and pathway level. The conclusion is consistent with the prediction of overall survival, that predictive performance is cancer type dependent and in general GE data provides the best predictive performance on either gene or pathway level relative to CNV and SPM data. One limitation of our study is that the analysis results were generated on only TCGA data and the conclusions have not been validated in non-TCGA datasets. The reason why we focused on TCGA in this study is that TCGA is the largest and richest collection of multi-omics and clinical data on a large group of cancer patients spanning the most common types of human cancer. It is difficult to find a large-scale database besides TCGA to conduct a comprehensive validation for this pan-cancer and multi-omics study. Some validation for specific cohorts and specific omics data types could be conducted through an analysis of curated datasets from individual research studies. Considering that the goal of this study is to comprehensively compare multi-omics data and pathway predictors relative to gene predictors for cancer prognosis prediction, this specific validation is beyond the scope of this study. Although the validation on other datasets besides TCGA is beyond the scope of our study, it is an important consideration and something we hope to explore in future work. One limitation of our study is that not all genes are included in our analysis because our analysis restricted the genes to include only the genes that are present in the Hallmark pathway collection to make a fair comparison between gene-level and pathway-level models. The models may fail to explore some genes that have a true association with patient survival. Subsequently, the definition of pathways in existing databases could also affect the performance. When gene level predictors are the only focus of interest, this restriction could be released to retain the full performance of prediction. Another limitation of our study is that we only fully explored three types of omics data (GE, CNV, SPM) and there are many omics data, which are not explored in our study, such as methylation data, miRNA data and proteomics data. For example, there are studies reporting the more stable prognostic power of methylation data relative to GE data on the univariable gene level [64]. We have conducted the basic analysis of using methylation data to predict the overall survival on both the gene and pathway level. Supplementary Figure S6 displays the comparison of concordance index values using single omics data on both the pathway and gene level. It shows that in general the methylation data provides similar predictive performance with gene expression data, better than CNV and SPM data. This is biological meaningful since methylation could regulate gene expression. Figure S7 displays the comparison of Fleiss Kappa values using single omics data on both the pathway and gene level. It is consistent with Fig. 4 that the pathway-level models have higher Kappa values than gene-level models, which indicates better robustness across multiple cross validation splits and replications. The methylation data is slightly less stable than gene expression data in our multivariable model on both the pathway and gene level. The detailed exploration on methylation data is beyond the scope of this study and may be explored in the future work.

## Conclusion

Based on this study, we found that predictive performance is cancer type dependent and, for the cohorts including GBM, LAML, PCPG, ESCA, PRAD, READ, TGCT, COAD and OV, all evaluated models have poor prognostic power. This finding implies that for these cancer types, more cancer specific clinical information should be used for model estimation in addition to multi-omics data to achieve significant predictive performance. For all other cohorts, we demonstrated that the pathway-level models have the advantages of better interpretation, higher stability and smaller model size, and in general GE data provides the best predictive performance on either gene or pathway level relative to CNV and SPM data. We also highlighted omics modalities that support the most accurate prognostic models. Beyond this, we showed that based on our results, the LGG, ACC and THCA cohorts are noteworthy. For the LGG cohort, all models have good predictive power with the SPM- having the best predictive performance (CI is 0.83). This implies that effective prognostic performance for this cohort can be achieved without gene expression data. For the THCA cohort, it is surprising that among all models, only the pathway-level SPM model can predict well.

## Supplementary Information

**Additional file 1.**

## Data Availability

The datasets analyzed during the current study are available in the UCSC TCGA repository, http://xena.ucsc.edu/, and MSigDB database, http://software.broadinstitute.org/gsea/msigdb/genesets.jsp?collection=H
