# Supplementary material for: Pan-cancer evaluation of gene expression and somatic alteration data for cancer prognosis prediction
Source: BMC Cancer. 2021 Sep 25;21:1053. doi: 10.1186/s12885-021-08796-3 (PMC8467202; doi:10.1186/s12885-021-08796-3)
Supplement: Supplementary file 1 — Additional file 1. [file 12885_2021_8796_MOESM1_ESM.pdf]

Pan-cancer evaluation of gene expression and somatic alteration data for cancer prognosis prediction

Xingyu Zheng, Christopher I. Amos, H. Robert Frost

List of Contents:

List of Tables ……………………………………………………………………………………… 2

X1 The full list of 33 cohorts analyzed in this study and some clinical characteristics. ……… 2

List of Figures ……………………………………………………………………………………. 4

S1 Comparative results of gene filtering. ……………………………………………………………………….. 4
S2 Comparative results of integration models with filtering or Group Lasso. ……………………. 6
S3 Comparative results of adjusting clinical stage in the model. ……………………………………….. 8
S4 Proportions of variable selection in the integration models. ……………………………………. 10
S5 Comparative results for Disease Free Interval outcome. ……………………………………………. 12
S6 Comparative results for methylation data. …………………………………………………………………… 14
S7 Comparative results of Fleiss Kappa values. ……………………………………………………………… 16

Table 1 The full list of 33 cohorts analyzed in this study and some clinical characteristics.

Cohort	Full name	Sample size GE	Sample size CNV	Sample size SPM	Death rate GE	Death rate CNV	Death rate SPM
ACC	Adrenocortical Carcinoma	79	89	90	0.35	0.36	0.37
BLCA	Bladder Carcinoma	403	404	391	0.44	0.44	0.45
BRCA	Breast Cancer	1080	1064	759	0.14	0.14	0.15
CESC	Cervical Cancer	290	282	196	0.24	0.24	0.21
COAD	Colon Cancer	275	428	210	0.25	0.22	0.23
COADREAD	Colon and Rectal Cancer	367	585	205	0.23	0.21	0.17
ESCA	Esophageal Cancer	184	184	183	0.42	0.41	0.41
GBM	Glioblastoma	152	570	277	0.76	0.81	0.75
GBMLGG	lower grade glioma and glioblastoma	663	1078	446	0.36	0.54	0.60
HNSC	Head and Neck Cancer	517	519	507	0.43	0.42	0.43
KICH	Kidney Chromophobe	65	65	65	0.14	0.14	0.14
KIRC	Kidney Clear Cell Carcinoma	531	526	213	0.33	0.33	0.25
KIRP	Kidney Papillary Cell Carcinoma	287	285	279	0.15	0.15	0.15
LAML	Acute Myeloid Leukemia	149	166	171	0.62	0.63	0.62
LGG	Lower Grade Glioma	511	508	511	0.24	0.25	0.24
LIHC	Liver Cancer	365	364	199	0.36	0.35	0.45
LUAD	Lung Adenocarcinoma	502	503	477	0.36	0.36	0.36
LUNG	Lung Cancer	996	997	338	0.40	0.40	0.44
LUSC	Lung Squamous Cell Carcinoma	494	494	175	0.43	0.43	0.49
MESO	Mesothelioma	85	85	81	0.86	0.86	0.85
OV	Ovarian Cancer	303	564	141	0.60	0.59	0.65

PAAD	Pancreatic Cancer	177	183	183	0.53	0.55	0.54
PCPG	Pheochromocytoma and Paraganglioma	177	160	177	0.03	0.04	0.03
PRAD	Prostate Cancer	496	491	497	0.02	0.02	0.02
READ	Rectal Cancer	92	157	80	0.18	0.17	0.20
SARC	Sarcoma	259	257	255	0.38	0.38	0.38
STAD	Stomach Cancer	103	413	355	0.28	0.40	0.37
TGCT	Testicular Cancer	387	134	134	0.40	0.03	0.03
THCA	Thyroid Cancer	134	498	495	0.03	0.03	0.03
THYM	Thymoma	504	122	122	0.03	0.07	0.07
UCEC	Endometroid Cancer	119	537	194	0.08	0.17	0.13
UVM	Ocular Melanoma	174	80	80	0.18	0.29	0.29

Figure S1 Comparative results of gene filtering using the output from a univariable Cox model for single omics models.

‘PLv’ represents ‘pathway-level’ and ‘GLv’ represents ‘gene-level’. The dots represent the values of the concordance index and the bars represent the standard error.

‘UniCox filter’ means we restricted the genes to include only the genes that are significant genes (p-value less than 0.05) in univariable Cox models.

For PLv SPM model, filtering resulted in a model without any pathway-level predictors at the optimal Lasso penalization threshold (the relative performance for this model is therefore not included in Supplementary Figure S1).

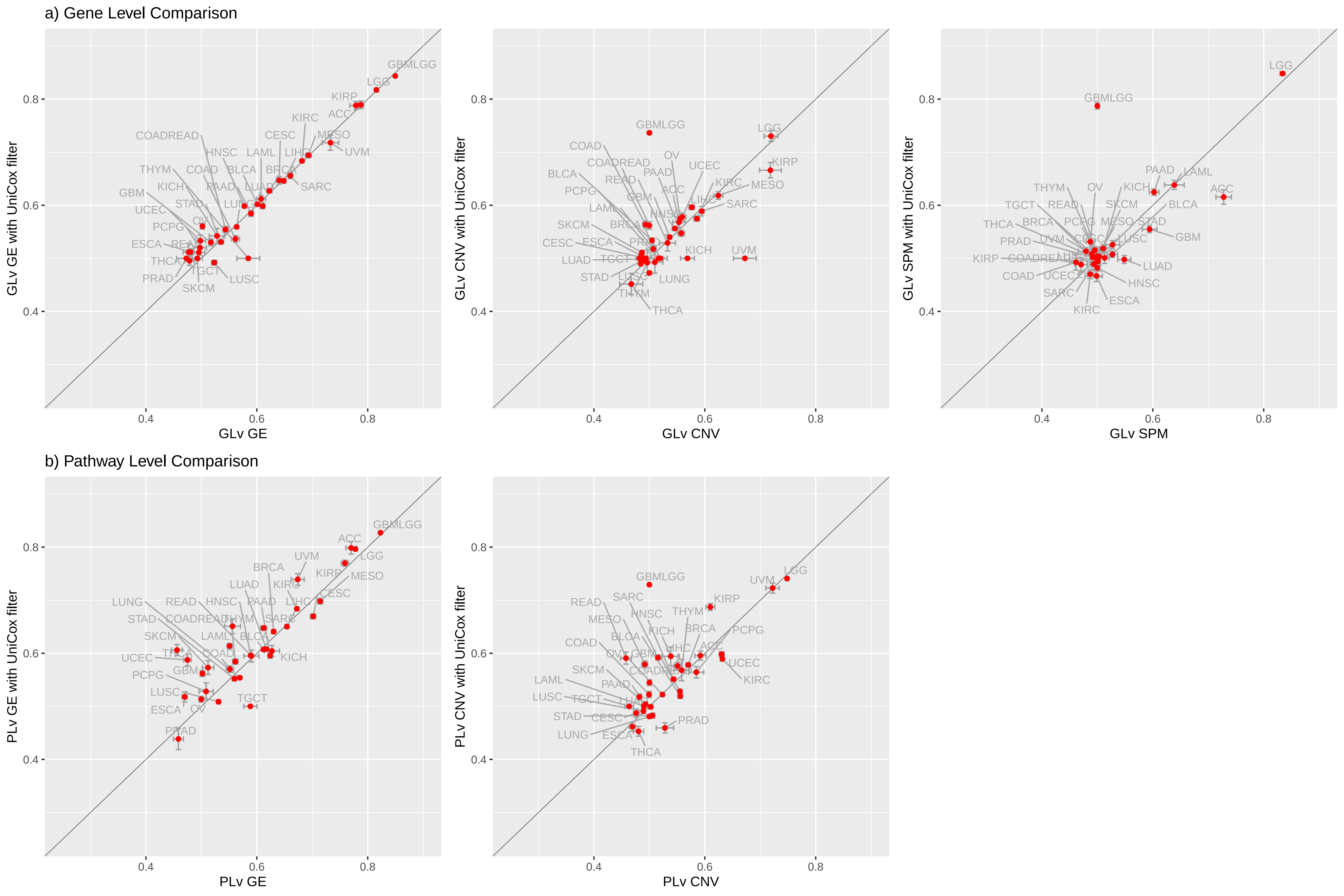

a) Gene Level Comparison
GLv GE with UniCox filter
GLv GE
GBMLGG
LGG
KIRP
ACC
KIRC
MESO
UVM
COADREAD
CESC
HNSC
LAML
LIHC
THYM
COAD
BLCA
BRCA
SARC
KICH
PAAD
LUAD
GBM
STAD
UCEC
OV
PCPG
ESCA
READ
THCA
TGCT
LUSC
PRAD
SKCM
0.4
0.6
0.8
GLv CNV with UniCox filter
GLv CNV
GBMLGG
LGG
COAD
COADREAD
OV
UCEC
KIRP
BLCA
PAAD
READ
KIRC
MESO
PCPG
ACC
GBM
LIHC
SARC
LAML
HNSC
SKCM
BRCA
CESC
ESCA
PRAD
KICH
UVM
LUAD
TGCT
STAD
LUSC
LUNG
THYM
THCA
GLv SPM with UniCox filter
GLv SPM
LGG
GBMLGG
PAAD
LAML
THYM
OV
KICH
ACC
TGCT
READ
SKCM
BLCA
THCA
BRCA
PCPG
MESO
STAD
PRAD
UVM
CESC
LUSC
GBM
KIRP
COADREAD
LUAD
COAD
UCEC
HNSC
SARC
ESCA
KIRC
b) Pathway Level Comparison
PLv GE with UniCox filter
PLv GE
GBMLGG
ACC
UVM
LGG
BRCA
KIRP
MESO
LUAD
KIRC
CESC
LUNG
READ
HNSC
PAAD
LIHC
STAD
COADREAD
THYM
SARC
SKCM
LAML
BLCA
COAD
KICH
UCEC
THCA
GBM
PCPG
LUSC
TGCT
ESCA
OV
PRAD
PLv CNV with UniCox filter
PLv CNV
GBMLGG
UVM
LGG
READ
SARC
KIRP
MESO
HNSC
THYM
BLCA
KICH
BRCA
PCPG
COAD
OV
GBM
LIHC
ACC
UCEC
SKCM
COADREAD
KIRC
LAML
PAAD
LUSC
TGCT
STAD
CESC
PRAD
LUNG
ESCA
THCA

Figure S2 Comparative results of integration models with filtering or Group Lasso.

'PLv' represents 'pathway-level' and 'GLv' represents 'gene-level'. The dots represent the values of the concordance index and the bars represent the standard error.

'UniCox filter' means we restricted the genes to include only the genes that are significant genes (p-value less than 0.05) in univariable Cox models.

'Cosmic filter' means we restricted the genes to include only the genes that are present in the Cosmic database.

For row a and b, we implemented the first integrative method as described in Methods section, which is combination. For row c, we implemented the second integrative method as described in Methods section, which is Group Lasso.

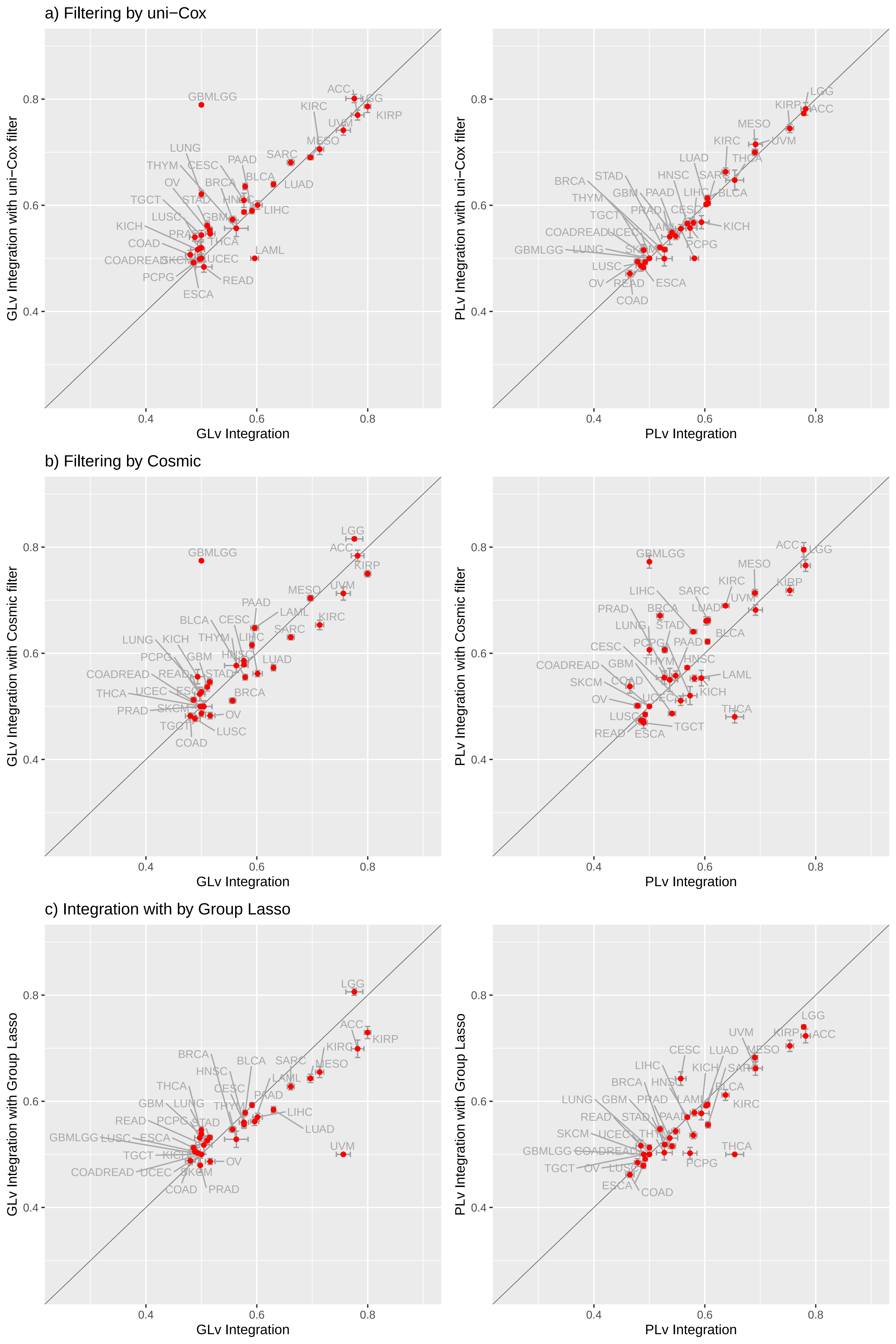

a) Filtering by uni−Cox
GLv Integration with uni−Cox filter
GLv Integration
PLv Integration with uni−Cox filter
PLv Integration
b) Filtering by Cosmic
GLv Integration with Cosmic filter
GLv Integration
PLv Integration with Cosmic filter
PLv Integration
c) Integration with by Group Lasso
GLv Integration with Group Lasso
GLv Integration
PLv Integration with Group Lasso
PLv Integration
0.4
0.6
0.8

Figure S3 Comparative results of adjusting clinical stage in the model.

‘PLv’ represents ‘pathway-level’ and ‘GLv’ represents ‘gene-level’. The dots represent the values of the concordance index and the bars represent the standard error.

‘stage adjustment’ means that we added the adjustment of clinical stage variable in the models.

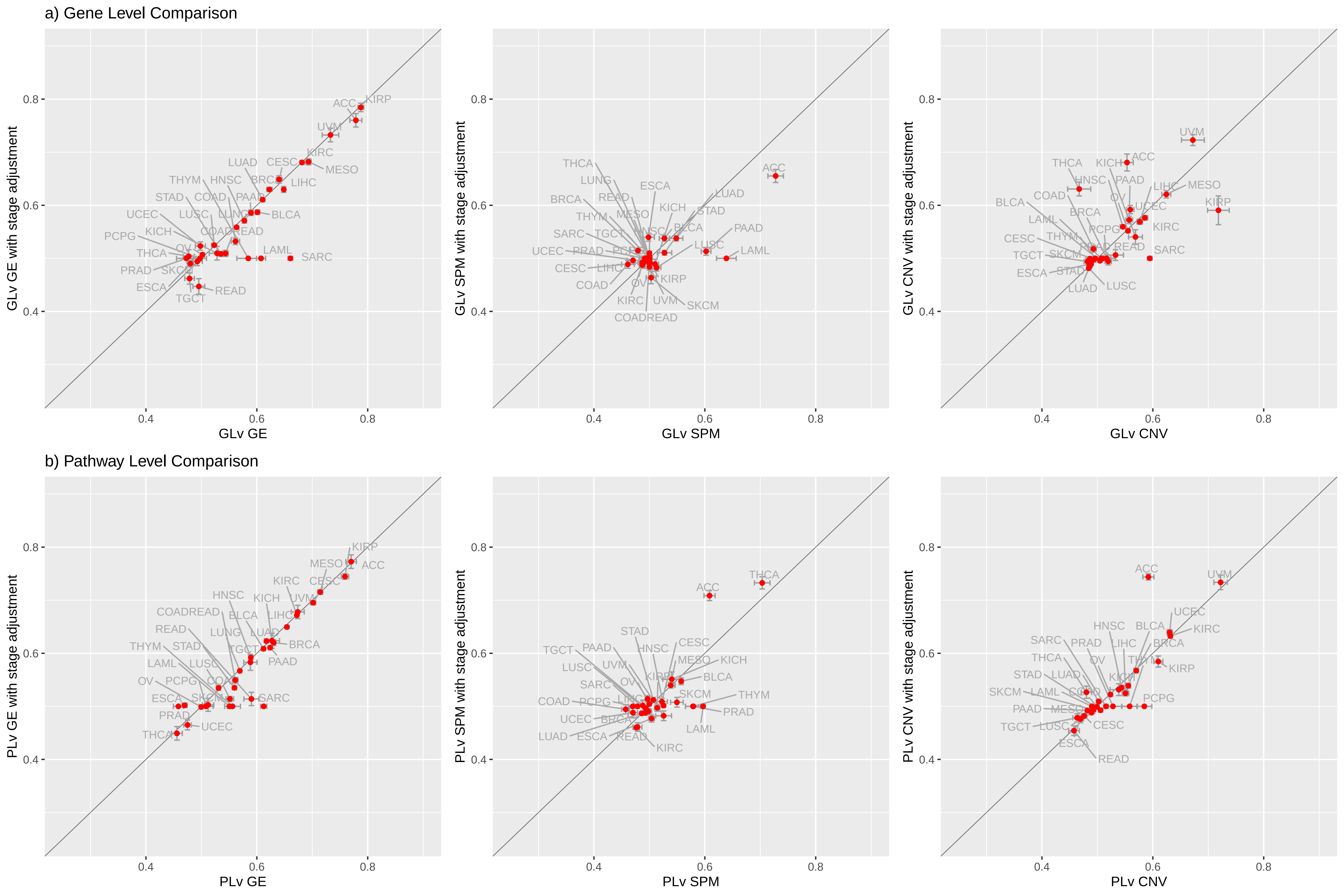

a) Gene Level Comparison
GLv GE with stage adjustment
GLv GE
GLv SPM with stage adjustment
GLv SPM
GLv CNV with stage adjustment
GLv CNV
b) Pathway Level Comparison
PLv GE with stage adjustment
PLv GE
PLv SPM with stage adjustment
PLv SPM
PLv CNV with stage adjustment
PLv CNV
0.4
0.6
0.8

Figure S4 Proportions of variable selection for single type of data in the integration models.

These heatmaps display the values of the proportions of each type of variable in the integrative models (GE, CNV and SPM separately), which are between 0 and 1.

'PLv' represents 'pathway-level' and 'GLv' represents 'gene-level'.

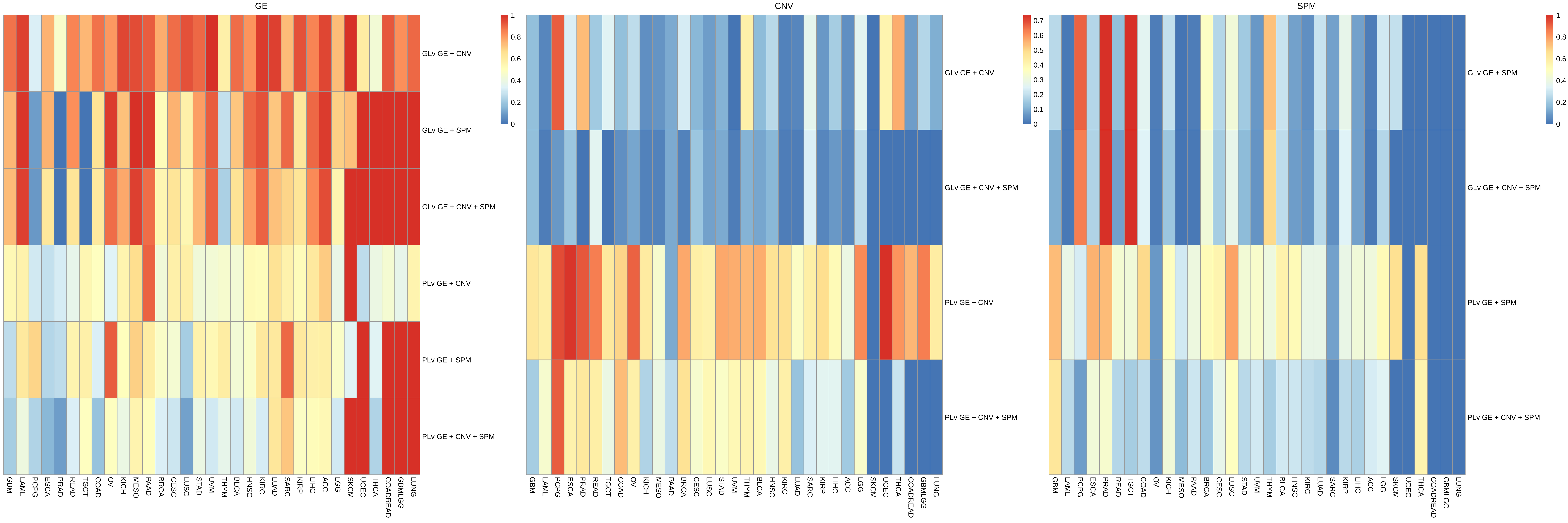

GE
CNV
SPM
GLv GE + CNV
GLv GE + SPM
GLv GE + CNV + SPM
PLv GE + CNV
PLv GE + SPM
PLv GE + CNV + SPM
GLv GE + CNV
GLv GE + CNV + SPM
PLv GE + CNV
PLv GE + CNV + SPM
GLv GE + SPM
GLv GE + CNV + SPM
PLv GE + SPM
PLv GE + CNV + SPM
1
0.8
0.6
0.4
0.2
0
0.7
0.6
0.5
0.4
0.3
0.2
0.1
0
GBM LAML PCPG ESCA PRAD READ TGCT COAD OV KICH MESO PAAD BRCA CESC LUSC STAD UVM THYM BLCA HNSC KIRC LUAD SARC KIRP LIHC ACC LGG SKCM UCEC THCA COADREAD GBMLGG LUNG
GBM LAML PCPG ESCA PRAD READ TGCT COAD OV KICH MESO PAAD BRCA CESC LUSC STAD UVM THYM BLCA HNSC KIRC LUAD SARC KIRP LIHC ACC LGG SKCM UCEC THCA COADREAD GBMLGG LUNG
GBM LAML PCPG ESCA PRAD READ TGCT COAD OV KICH MESO PAAD BRCA CESC LUSC STAD UVM THYM BLCA HNSC KIRC LUAD SARC KIRP LIHC ACC LGG SKCM UCEC THCA COADREAD GBMLGG LUNG

Figure S5 Comparative results of predicting Disease Free Interval outcome for both gene-level and pathway-level prognostic models estimated using GE, SPM and CNV data from multiple cancer types.

‘PLv’ represents ‘pathway-level’ and ‘GLv’ represents ‘gene-level’. The dots represent the values of the concordance index and the bars represent the standard error.

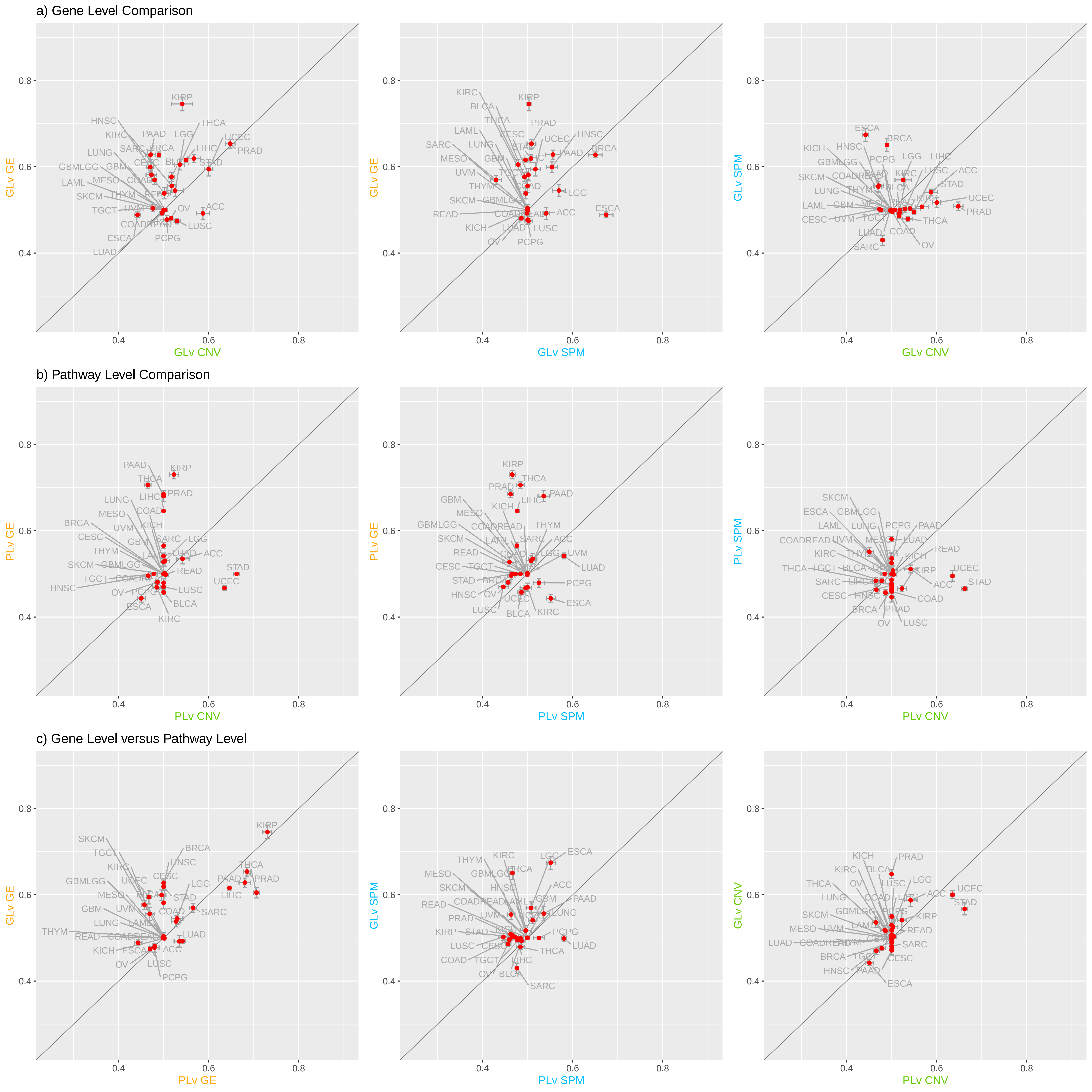

a) Gene Level Comparison
GLv GE
GLv CNV
GLv SPM
b) Pathway Level Comparison
PLv GE
PLv CNV
PLv SPM
c) Gene Level versus Pathway Level
0.4
0.6
0.8

Figure S6 Comparative results for both gene-level and pathway-level prognostic models estimated using GE, SPM, CNV and methylation data from multiple cancer types.

‘PLv’ represents ‘pathway-level’ and ‘GLv’ represents ‘gene-level’. The dots represent the values of the concordance index and the bars represent the standard error.

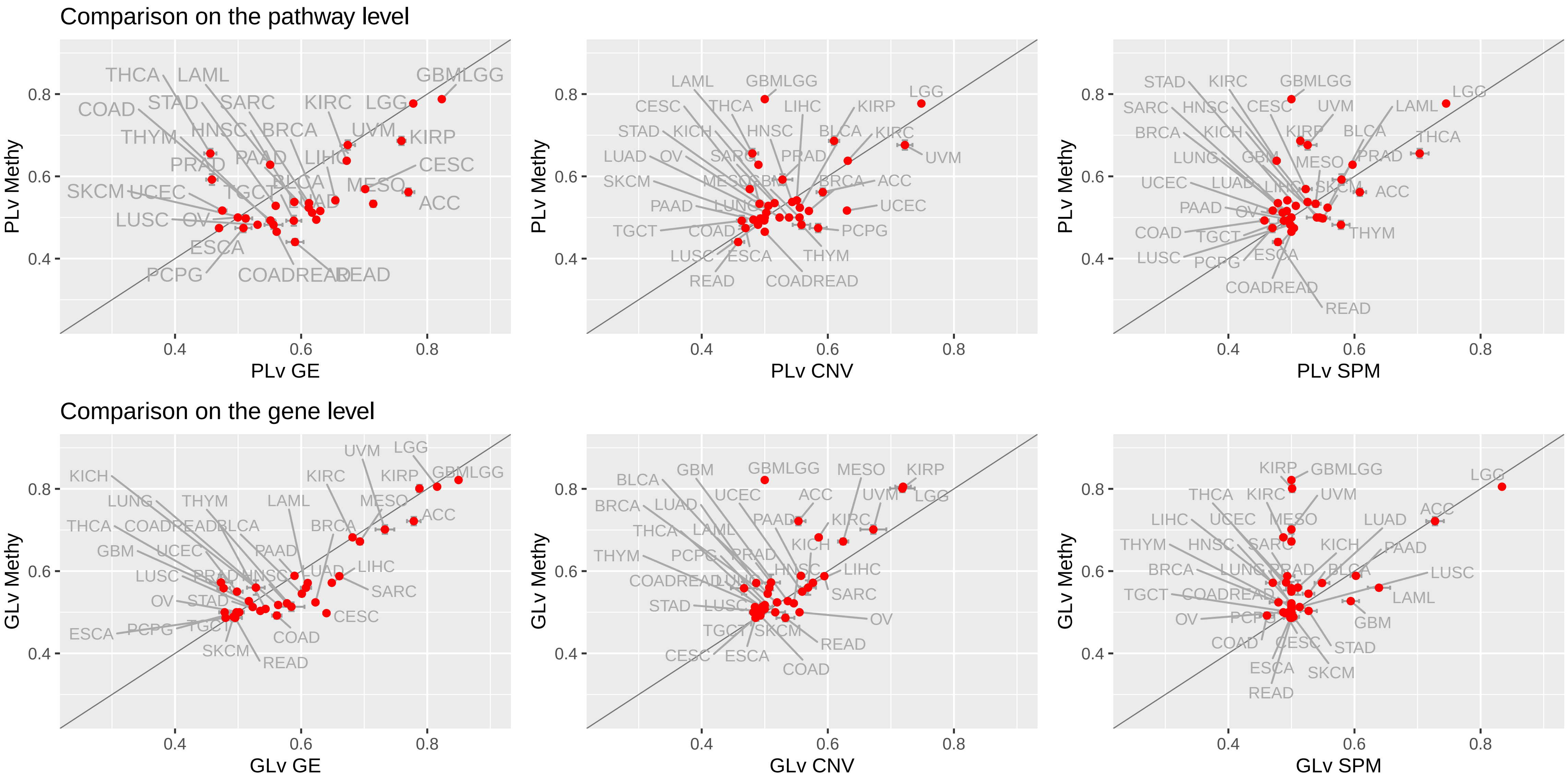
Comparison on the pathway level
PLv Methy
PLv GE
PLv CNV
PLv SPM
0.4
0.6
0.8
Comparison on the gene level
GLv Methy
GLv GE
GLv CNV
GLv SPM

Figure S7 Heatmap of Fleiss Kappa statistics across cohorts and models.

‘PLv’ represents ‘pathway-level’ and ‘GLv’ represents ‘gene-level’. The cells in grey represent models that cannot converge and in this case, no predictors could be selected to predict prognosis.

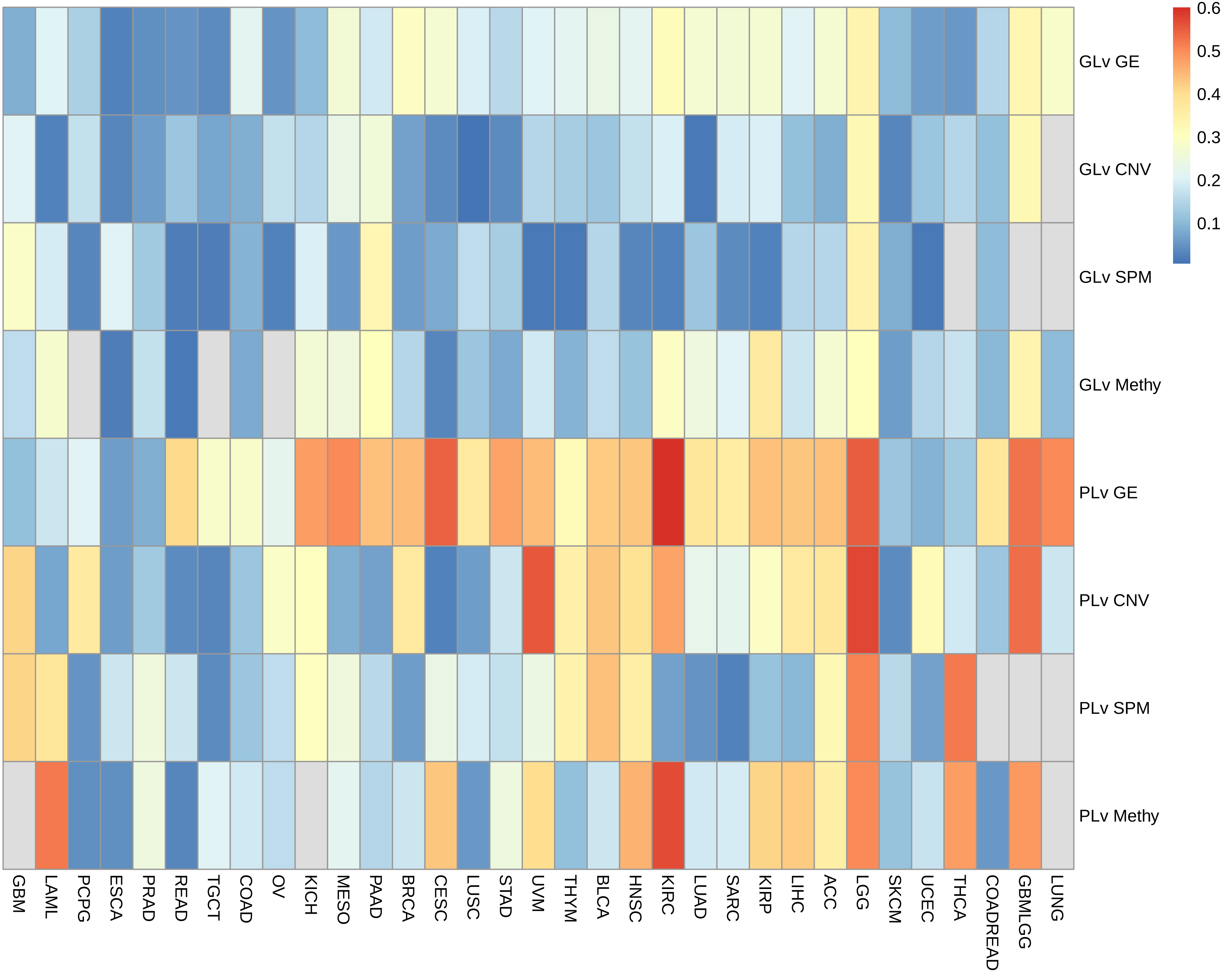

0.6
0.5
0.4
0.3
0.2
0.1
GLv GE
GLv CNV
GLv SPM
GLv Methy
PLv GE
PLv CNV
PLv SPM
PLv Methy
GBM
LAML
PCPG
ESCA
PRAD
READ
TGCT
COAD
OV
KICH
MESO
PAAD
BRCA
CESC
LUSC
STAD
UVM
THYM
BLCA
HNSC
KIRC
LUAD
SARC
KIRP
LIHC
ACC
LGG
SKCM
UCEC
THCA
COADREAD
GBMLGG
LUNG
